# Mitochondrial Methionyl-tRNA Formyltransferase Deficiency Alleviates Metaflammation by Modulating Mitochondrial Activity in Mice

**DOI:** 10.3390/ijms24065999

**Published:** 2023-03-22

**Authors:** Xiaoxiao Sun, Suyuan Liu, Jiangxue Cai, Miaoxin Yang, Chenxuan Li, Meiling Tan, Bin He

**Affiliations:** 1 Key Laboratory of Animal Physiology & Biochemistry, Ministry of Agriculture and Rural Affairs, College of Veterinary Medicine, Nanjing Agricultural University, Nanjing 210095, China; 2 MOE Joint International Research Laboratory of Animal Health & Food Safety, Nanjing Agricultural University, Nanjing 210095, China

**Keywords:** Mitochondrial methionyl-tRNA formyltranse (Mtfmt), mitochondria, mitochondrial reactive oxygen species (mROS), metaflammation, metabolic disorder

## Abstract

Various studies have revealed the association of metabolic diseases with inflammation. Mitochondria are key organelles involved in metabolic regulation and important drivers of inflammation. However, it is uncertain whether the inhibition of mitochondrial protein translation results in the development of metabolic diseases, such that the metabolic benefits related to the inhibition of mitochondrial activity remain unclear. Mitochondrial methionyl-tRNA formyltransferase (Mtfmt) functions in the early stages of mitochondrial translation. In this study, we reveal that feeding with a high-fat diet led to the upregulation of Mtfmt in the livers of mice and that a negative correlation existed between hepatic *Mtfmt* gene expression and fasting blood glucose levels. A knockout mouse model of *Mtfmt* was generated to explore its possible role in metabolic diseases and its underlying molecular mechanisms. Homozygous knockout mice experienced embryonic lethality, but heterozygous knockout mice showed a global reduction in Mtfmt expression and activity. Moreover, heterozygous mice showed increased glucose tolerance and reduced inflammation, which effects were induced by the high-fat diet. The cellular assays showed that *Mtfmt* deficiency reduced mitochondrial activity and the production of mitochondrial reactive oxygen species and blunted nuclear factor-κB activation, which, in turn, downregulated inflammation in macrophages. The results of this study indicate that targeting *Mtfmt*-mediated mitochondrial protein translation to regulate inflammation might provide a potential therapeutic strategy for metabolic diseases.

## 1. Introduction

Type 2 diabetes (T2D) is becoming an increasingly prevalent medical and economic burden worldwide [1,2]. Many studies have revealed the association of metabolic diseases with inflammation, and at present metabolic diseases caused by chronic low-grade inflammation are defined as metaflammation [3,4,5,6]. Metaflammation is an inflammatory complication of metabolic disorders characterized by altered levels of inflammatory cytokines, adipokines, and lipid mediators [7]. Metaflammation, caused by immune cells, such as macrophages living in or infiltrating metabolic organs under obesity, impairs insulin action and results in insulin resistance [8]. In recent studies, obesity has been associated with increased secretion of monocyte chemoattractant protein-1 from adipocytes, which results in a higher number of infiltrating adipose tissue macrophages. The chemokines and cytokines secreted by infiltrating macrophages enhance local inflammatory responses and affect gene expression in adipocytes, resulting in insulin resistance systemically [9]. In addition, diet-induced hepatic steatosis and insulin resistance are prevented by the depletion of Kupffer cells (liver-specific macrophages) [10].

Mitochondria are key organelles involved in metabolic regulation, and their dysfunction is closely associated with metabolic diseases [11,12]. In obesity and insulin resistance, inhibition of mitochondrial electron transport chain (ETC) activity has been shown to have metabolic benefits [13,14]. The ETC uses a series of electron transfer reactions to generate cellular adenosine triphosphate via oxidative phosphorylation (OxPhos). Mitochondrial reactive oxygen species (mROS) are generated because of electron transfer [15]. Moderate levels of mROS are necessary for cell signaling and organismal health, but high levels of mROS result in damage to the body [16,17]. Thioredoxin 2 (*Trx2*) deletion in adipose tissue leads to an increase in mROS, which then contributes to increased secretion of systemic inflammatory factors via the activation of nuclear factor-κB (NFκB), resulting in the disruption of systemic glucose and lipid metabolism [18]. On the contrary, reducing mROS output alleviates high-fat-diet-induced cirrhosis and insulin resistance [19]. Metformin, a widely used drug for treating T2D, has been shown to delay diabetes and vascular dysfunction in rats by slowing mROS production [20].

In mammalian mitochondria, 13 proteins are synthesized that are essential subunits of oxidative phosphorylation [21]. It has been well established that the impaired translation of mitochondrial proteins leads to mitochondrial disorders and consequently affects organismal health [22]. Mitochondrial protein translation is similar to bacterial protein translation in that it involves the initiation, elongation, and termination of translation. However, the initiation of mammalian mitochondrial protein translation is different in that bacteria encode two different tRNA^Met^s involved in initiation and elongation, whereas mammalian mitochondria encode only one tRNA^Met^ that performs both functions. During mitochondrial protein translation, methionyl-tRNA formyltransferase (Mtfmt) formylates a part of Met-tRNA^Met^ after it has been aminoacylated to initiate mitochondrial protein translation [23]. Studies have shown that *Mtfmt* double heterozygous mutation could lead to a series of visual, neurological, and muscular impairments and multiple mitochondrial respiratory chain deficiencies [24,25,26]. However, the involvement of *Mtfmt*-mediated mitochondrial protein translation in the development of metabolic diseases remains unclear.

The purpose of the present study was to determine whether *Mtfmt* plays an important role in metabolic inflammation, as well as to explore the mechanisms underpinning its development. The heterozygous knockout mice displayed decreased Mtfmt protein expression as well as high-fat-diet (HFD)-induced inflammation, despite the homozygous knockout mice exhibiting embryonic lethality. The cellular assays revealed that the *Mtfmt* knockdown of macrophages in vitro reduced mROS production and NFκB activation, which in turn affected inflammation. These data demonstrated that targeting *Mtfmt*-mediated mitochondrial protein translation to regulate levels of mROS might be an innovative therapeutic option for treating metabolic diseases.

## 2. Results

### 2.1. Expression Levels of Mtfmt Were Correlated with Diet and Glucose Levels

**Figure 1:**
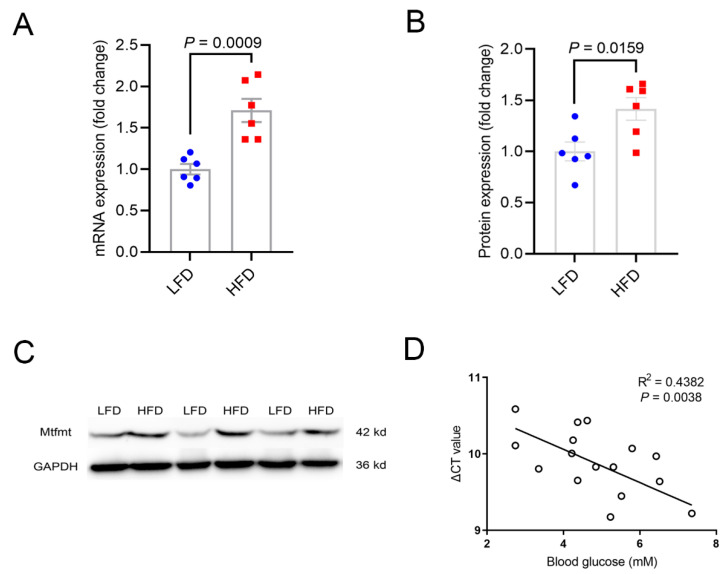
Expression levels of mitochondrial methionyl-tRNA formyltranse (*Mtfmt*) were correlated with diet and glucose levels. (**A**,**B**) Expression of Mtfmt in the livers of low-fat-diet (LFD) and high-fat-diet (HFD) male mice. (**C**) Mtfmt protein expression in the livers of LFD and HFD male mice. (**D**) Correlation analysis between *Mtfmt* gene expression and fasting glucose levels.

We compared *Mtfmt* expression in the livers of mice fed an LFD with the expression levels of mice fed an HFD to investigate the correlation between *Mtfmt* and metabolic states. The abundances of Mtfmt mRNA and proteins were significantly higher in high-fat-diet (HFD, 60% fat)-fed compared with low-fat-diet (LFD, 10% fat)-fed mice (Figure 1A–C). Further, the expression of *Mtfmt* in the liver was negatively correlated with fasting glucose levels (Figure 1D).

### 2.2. Mtfmt Deletion Caused Embryonic Lethality in Mice

**Figure 2:**
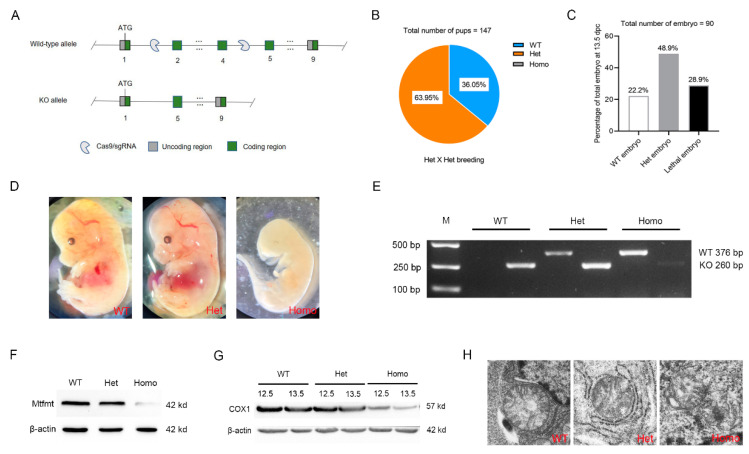
*Mtfmt* deletion caused embryonic lethality in mice. (**A**) Disruption of *Mtfmt* in ES cells by CRISPR-Cas9 technology. (**B**) Percentages of different genotypes of the pups from Het × Het breeding. WT: wild type, Het: heterozygous, Homo: homozygous. The total number of pups analyzed was 147. (**C**) The percentages of embryos with different genotypes at 13.5 dpc. The total number of embryos analyzed was 90. (**D**) Representative images of Homo, Het, and WT embryos. (**E**) Representative genotyping results generated by PCR. Genomic DNA was isolated from mouse embryos. KO mutation can be amplified as a band of 260 bp; WT can be amplified as a band of 376 bp. (**F**) Representative Western blots of Mtfmt and β-actin expression in the embryos. (**G**) Representative Western blots of COX1 and β-actin expression in the embryos at 12.5 dpc and 13.5 dpc. (**H**) Representative images of mitochondria in Homo, Het, and WT embryos (scale bar, 500 nm).

The *Mtfmt* knockout mouse model was constructed to investigate a specific and causal role of *Mtfmt* deficiency in the development of metabolic states (Figure 2A). The genotyping results from the Het × Het breeding indicated that, out of 147 surviving mice, 94 (63.95%) were Het KO mice and 53 (36.05%) were wild-type (WT) mice (Figure 2B). Embryos from 9.5 to 13.5 dpc were collected for genotyping to further validate this observation. A total of 90 embryos were collected, of which 26 died prematurely, and all of them were Homo KO mice (i.e., 28.9% of all embryos died prematurely). Further, out of the 64 surviving embryos, 20 (22.2%) and 44 (48.9%) embryos were WT and Het KO types, respectively (Figure 2C). A significant developmental delay was observed in the Homo KO embryos at 13.5 dpc compared with the WT and Het KO embryos (Figure 2D,E). However, no significant difference was observed between the Het KO and WT embryos (Figure 2D,E). Western blot results showed that the Mtfmt protein was barely detectable in Homo KO embryos (as some of the embryos might have had maternal tissue contamination) (Figure 2F). Since *Mtfmt* is involved in the initiation of mitochondrial protein translation, we hypothesized that the knockdown of *Mtfmt* would affect mitochondrial protein translation and thus mitochondrial morphology and function. The abundance of the COX1 protein, which is encoded by mitochondrial DNA (mtDNA), was significantly lower in Homo KO embryos at 13.5 dpc than at 12.5 dpc (Figure 2G). Transmission electron microscopy results showed mitochondrial swelling, shortened ridges, and ruptured mitochondrial membranes in Homo KO embryos, while slightly swollen mitochondria were observed in the Het KO embryos compared with the WT embryos (Figure 2H). Hence, these results indicated that the complete *Mtfmt* knockout resulted in embryonic lethality. Moreover, the knockout affected mitochondrial protein translation and thus mitochondrial morphology, whereas Het KO mice appeared to be normal in the basal condition.

### 2.3. Mtfmt Haploinsufficiency Improved Hepatic Metabolic Health

**Figure 3:**
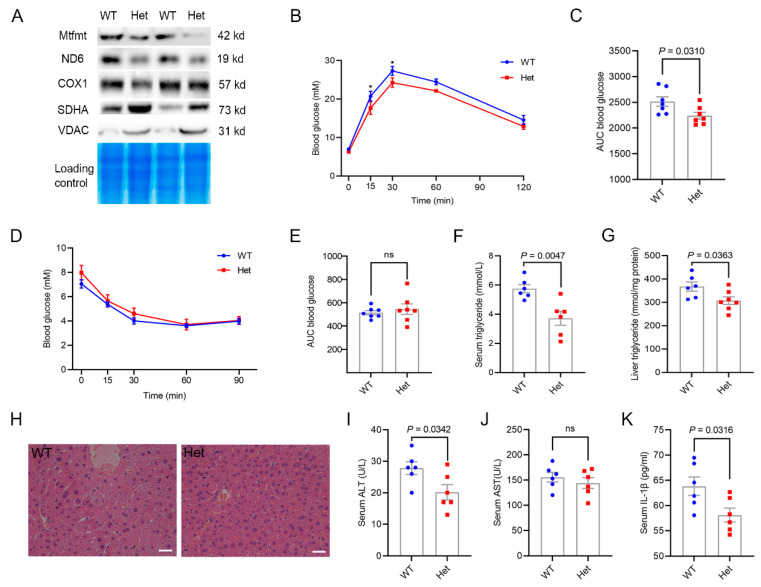
*Mtfmt* haploinsufficiency improved hepatic metabolic health. (**A**) The expression of Mtfmt, ND6, COX1, SDHA, and VDAC in the liver mitochondria of WT and Het male mice. (**B**) Blood glucose levels as assayed by GTT of 23-week-old Het and WT male mice, * *p* < 0.05 (WT versus Het). (**C**) Area under the curve (AUC) statistics for GTT, *n* = 7. (**D**) Blood glucose levels as assayed by ITT of 23-week-old Het and WT male mice. (**E**) AUC statistics for ITT, *n* = 7, ns indicates no significance. (**F**) Blood triglyceride concentrations in 23-week-old Het and WT male mice. (**G**) Liver triglyceride concentrations in 23-week-old Het and WT male mice. (**H**) Representative images of H&E-stained sections of livers from 23-week-old Het and WT male mice (scale bar, 20 μm). (**I**,**J**) Blood ALT (**I**) and AST (**J**) concentrations in 23-week-old Het and WT male mice, ns indicates no significance. (**K**) Blood IL-1β concentrations in 23-week-old Het and WT male mice.

The abundances of the Mtfmt protein in liver mitochondria were detected, and the results showed a slight downregulation of the Mtfmt protein in the liver of the Het KO mice compared with the WT mice (Figure 3A). In addition, the abundances of the ND6 and COX1 proteins, which are encoded by mtDNA, were significantly lower in the Het KO mice compared with the WT mice (Figure 3A). Interestingly, the abundances of succinate dehydrogenase complex subunit A (SDHA) and voltage-dependent anion channels (VDACs), which are encoded by nuclear DNA and transported to mitochondria, were higher in the Het KO mice compared with the WT mice (Figure 3A).

As the expression of *Mtfmt* in the liver is negatively correlated with fasting glucose levels, the glucose tolerance test (GTT) and insulin tolerance test (ITT) were performed on WT and Het KO male mice at 23 weeks of age. The GTT results showed a slight but significant increase in glucose clearance in the Het KO mice compared with the WT mice (Figure 3B,C). No difference was observed in the ITT results (Figure 3D,E). Triglyceride contents in the serum (Figure 3F) and liver (Figure 3G) were significantly lower in the Het KO mice compared with the WT mice. Hematoxylin-eosin (H&E) staining of the liver showed reduced liver lipid deposition in Het KO mice (Figure 3H). The levels of alanine transaminase, but not aspartate transaminase, were significantly lower in Het KO mice (Figure 3I,J). Previous studies showed that interleukin (IL)-1β in human blood is positively correlated with insulin resistance [27]. In this study, the IL-1β levels were significantly lower in the serum of the Het KO mice compared with the WT mice (Figure 3K). Thus, these findings suggest that *Mtfmt* haploinsufficiency might improve hepatic metabolic health in mice.

### 2.4. Mtfmt Haploinsufficiency Alleviated HFD-Induced Metabolic Disorders

**Figure 4:**
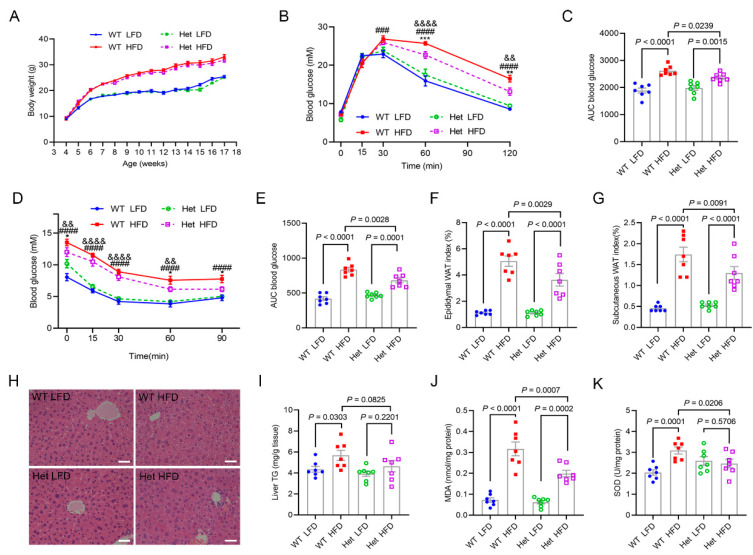
*Mtfmt* haploinsufficiency alleviates HFD-induced metabolic disorders. (**A**) Growth curves for WT LFD, WT HFD, Het LFD, and Het HFD mice. (**B**) Blood glucose levels as assayed by GTT. ** *p* < 0.01, *** *p* < 0.001 (WT HFD versus Het HFD); ^&&^ *p* < 0.01, ^&&&&^ *p* < 0.0001 (Het LFD versus Het HFD); ^###^ *p* < 0.001, ^####^ *p* < 0.0001 (WT LFD versus WT HFD). (**C**) Area under the curve (AUC) statistics for GTT. (**D**) Blood glucose levels as assayed by ITT. * *p* < 0.05 (WT HFD versus Het HFD); ^&&^ *p* < 0.01, ^&&&&^ *p* < 0.0001 (Het LFD versus Het HFD); ^####^ *p* < 0.0001 (WT LFD versus WT HFD). (**E**) AUC statistics for ITT. (**F**,**G**) Epididymal fat indexes and subcutaneous fat indexes (g). (**H**) Representative images of H&E-stained sections of livers (scale bar, 20 μm). (**I**) Determinations of triglyceride contents in livers. (**J**) Determinations of MDA contents in livers. (**K**) Determinations of SOD activity in liver.

Four-week-old WT and Het male mice were randomly divided into four groups to test whether *Mtfmt* haploinsufficiency could alleviate metabolic disorders: WT and Het male mice were fed a high-fat diet (HFD, 60% fat; WT HFD and Het HFD, respectively) or a low-fat diet (LFD, 10% fat; WT LFD and Het LFD, respectively). After feeding of the HFD for 17 weeks, the body weights of the WT HFD mice were significantly higher than those of the WT LFD mice (Figure 4A). No significant differences were observed between the body weights of the Het HFD mice and those of the WT HFD mice (Figure 4A). WT HFD mice exhibited increased blood glucose levels after chronic HFD during GTT (Figure 4B,C). On the contrary, the glucose levels were lower in the Het HFD mice compared with the WT HFD mice (Figure 4B,C). Moreover, the ITT results showed that glucose levels were significantly higher in the HFD group compared with the LFD group, while a significant increase in insulin sensitivity was observed in the Het HFD mice compared with the WT HFD mice (Figure 4D,E). The weights of epididymal fat (Figure 4F) and subcutaneous fat (Figure 4G) were significantly lower in the Het HFD mice compared with the WT HFD mice. H&E staining revealed that the Het HFD mice had significantly lower fat levels than the WT HFD mice (Figure 4H). Triglyceride contents were significantly lower in the livers of the Het HFD mice compared with the WT HFD mice (Figure 4I). Oxidative stress levels in the liver were assessed. Malondialdehyde levels were significantly lower in the Het HFD mice compared with the WT HFD mice (Figure 4J), but superoxide dismutase activity was not affected (Figure 4K). Hence, the data indicated that *Mtfmt* haploinsufficiency might protect against HFD-induced metabolic disorders.

### 2.5. Mtfmt Haploinsufficiency Alleviated HFD-Induced Inflammation

**Figure 5:**
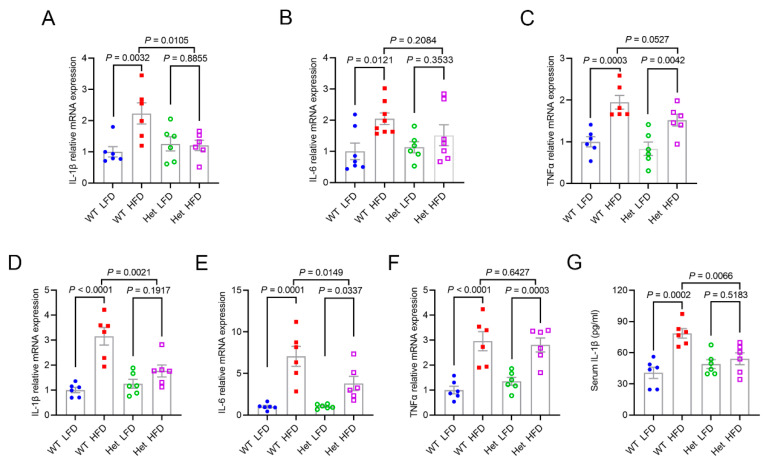
*Mtfmt* haploinsufficiency alleviates HFD-induced inflammation. (**A**–**C**) The relative expression levels of *IL-1β* (**A**), *IL-6* (**B**), and *TNFα* (**C**) mRNA in the livers of WT LFD, WT HFD, Het LFD, and Het HFD mice. (**D**–**F**) The relative expression levels of *IL-1β* (**D**), *IL-6* (**E**), and *TNFα* (**F**) mRNA in the epididymal fat of WT LFD, WT HFD, Het LFD, and Het HFD mice. (**G**) Blood IL-1β concentrations in WT LFD, WT HFD, Het LFD, and Het HFD mice.

We next detected the expression of pro-inflammatory factors *IL-1β*, *IL-6*, and tumor necrosis factor-*α* (*TNFα*) in the liver and epididymal fat. *IL-1β*, *IL-6*, and *TNFα* mRNAs were upregulated in the livers (Figure 5A–C) and epididymal fat (Figure 5D–F) of the WT HFD mice compared with the WT LFD mice. However, *IL-1β* and *IL-6* expression were significantly downregulated in the livers (Figure 5A,B) and epididymal fat (Figure 5D,E) of the Het HFD mice compared with the WT HFD mice. Moreover, the IL-1β levels in the serum of mice further indicated that *Mtfmt* haploinsufficiency alleviated HFD-induced inflammation (Figure 5G).

### 2.6. Mtfmt Knockdown in Macrophages Decreased Mitochondrial Activity and mROS Signaling and Blunted NFκB Signaling

**Figure 6:**
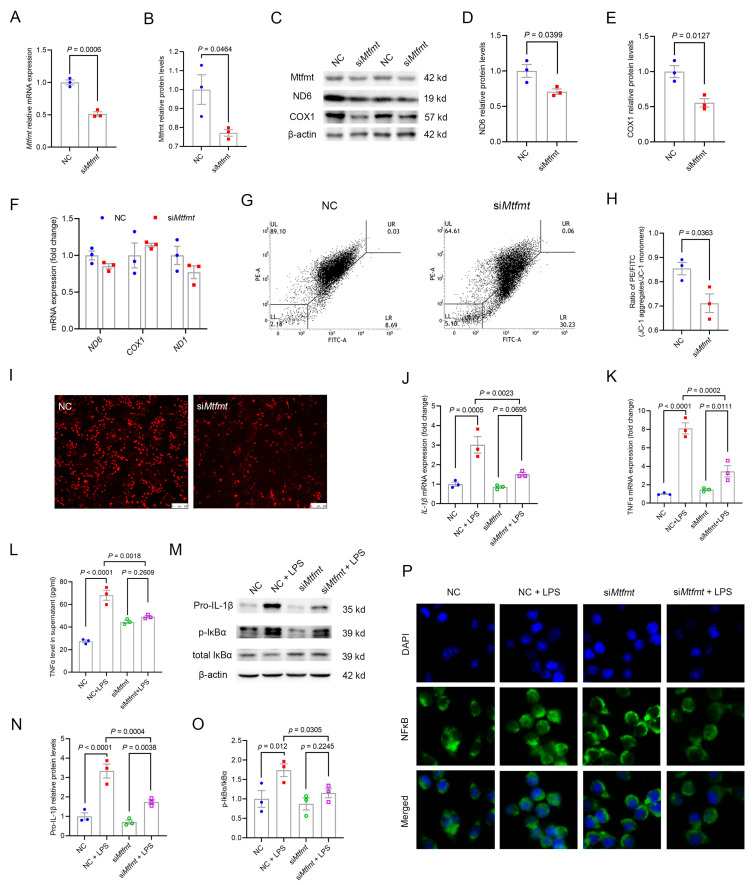
*Mtfmt* knockdown in macrophages decreased mitochondrial activity and mROS signaling and blunted NFκB signaling. *Mtfmt* siRNA was transfected into Kupffer cells for 24 h. (**A**) The mRNA levels of *Mtfmt* were determined by quantitative real-time PCR. (**B**) Histogram showing quantification of Mtfmt protein levels. (**C**) Protein levels of Mtfmt ND6 and COX1 were determined by Western blot analysis. (**D**) Histogram showing quantification of ND6 protein levels. (**E**) Histogram showing quantification of COX1 protein levels. (**F**) mRNA levels of mtDNA-encoded genes. (**G**) Kupffer cells were transfected with control or *Mtfmt* siRNA and stained with JC-1 to measure mitochondrial membrane potential by flow cytometric analysis. (**H**) Histogram showing rates of high mitochondrial membrane potential cells. (**I**) Kupffer cells were transfected with control or *Mtfmt* siRNA and stained with MitoSOX to measure mROS (scale bar, 100 μm). (**J**,**K**) mRNA levels of *IL-1β* and *TNFα* were determined by quantitative real-time PCR. (**L**) TNFα levels in supernatants were measured by ELISA. (**M**) Protein levels of Pro-IL-1β, p-IκBα, and total IκBα were determined by Western blot analysis. (**N**) Histogram showing quantification of IL-1β protein levels. (**O**) Histogram showing quantification of p-IκBα and total IκBα levels. (**P**) Kupffer cells were transfected with control or *Mtfmt* siRNA and treated with 1 μg/mL LPS for 30 min, then analyzed for RelA/p65 localization by immunofluorescent staining. Nuclei were stained with DAPI (scale bar, 25 μm).

We used Kupffer cells, the macrophages that reside in the liver and are involved in the immune regulation of the liver, to investigate whether the in vitro knockdown of *Mtfmt* in macrophages could alleviate the onset of inflammation to gain insight into the attenuated metaflammatory phenotype. After the transfection of Kupffer cells with si*Mtfmt* for 24 h (Supplementary Figure S1A), quantitative polymerase chain reaction (qPCR) (Figure 6A) and Western blotting (Figure 6B,C) were used to verify knockdown efficiency. The abundances of the mtDNA-encoded proteins ND6 and COX1 were down-regulated by si*Mtfmt* transfection (Figure 6C–E), but the mRNAs were unaltered (Figure 6F). This supported our hypothesis that *Mtfmt* knockdown blocks mitochondrial translation. The mitochondrial membrane potential and mROS levels were examined after *Mtfmt* knockdown. The results showed that the *Mtfmt* knockdown in Kupffer cells decreased the mitochondrial membrane potential (Figure 6G,H). Changes in OxPhos should be reflected in modifications in mROS generation, so we detected mROS contents. As expected, the production of mROS decreased under basal conditions after *Mtfmt* knockdown (Figure 6I and Supplementary Figure S1B). 

After 24 h of si*Mtfmt* transfection, 100 ng/mL lipopolysaccharide (LPS) was used to treat Kupffer cells to further explore whether *Mtfmt* knockdown in Kupffer cells affected inflammation. The qPCR results showed that, compared with NC + LPS, *Mtfmt* knockdown after 6 h of treatment with LPS significantly decreased *IL-1β* and *TNFα* mRNA levels (Figure 6J,K). Meanwhile, *Mtfmt* knockdown also reduced the levels of TNFα in cell supernatants (Figure 6L). Furthermore, IL-1β precursor and p-IκBα protein levels in the *Mtfmt* knockdown cell treatment with LPS showed significant downregulation compared with NC + LPS (Figure 6M–O). A RelA/p65 nuclear translocation assay was performed to examine the alleviated inflammatory phenotype after *Mtfmt* knockdown and showed a significant reduction in RelA/p65 nuclear translocation after *Mtfmt* knockdown and treatment of Kupffer cells with LPS for 30 min (Figure 6P and Supplementary Figure S1C). Hence, these results suggested that *Mtfmt* deficiency in macrophages reduced mitochondrial activity and mROS production, which decreased NFκB activation and, in turn, affected inflammation.

## 3. Discussion

In this study, we have shown correlations between hepatic *Mtfmt* and metabolic states. We generated a novel strain of global *Mtfmt* knockout mice to explore the potential role of *Mtfmt* in metabolic diseases. *Mtfmt* knockout resulted in embryonic lethality, consistent with previous findings that interference with mitochondrial biogenesis led to embryonic death in animals [28,29,30,31,32,33,34]. However, heterozygous mice, which displayed mild mitochondrial dysfunction, exhibited increased glucose tolerance and reduced inflammation induced by HFD.

To maintain normal metabolism and health, mitochondrial function is essential [35,36]. There is a wide range of metabolic consequences associated with genetic diseases related to mitochondrial dysfunction. Obesity and type 2 diabetes are associated with mitochondrial ETC dysregulation [37,38]. Decreased OxPhos gene expression in skeletal muscle is associated with insulin resistance in humans [39]. Additionally, a variety of studies have linked reduced mitochondrial oxidative metabolism to insulin resistance in humans [40,41]. The data for two different mouse models in this study showed that in heterozygous knockout *Mtfmt* mice, a slight OxPhos deficit brought on by the downregulation of mitochondrial translation could result in a state of reduced adiposity and improved insulin sensitivity. It was found that these effects exactly coincided with the metabolic changes observed in mice with OxPhos defects caused by apoptosis-inducing factor deficiency, increased glucose utilization, and decreased lipid storage, for example [13]. In addition, a previous study indicated that mice with deletion of muscle-specific mitochondrial transcription factor A (TFAM), which controls the transcription of all mitochondrial encoded genes, did not develop insulin resistance [42]. Meanwhile, Vernochet et al. reported that adipose tissue TFAM-specific knockout mice exhibited decreased levels of mtDNA-encoded proteins and were protected from diet-induced insulin resistance, which results were similar to our findings [43]. Moreover, recent studies have shown that alternate-day fasting or *SDHAF4* knockout in the liver can drive systemic metabolic benefits by inhibiting the assembly of mitochondrial complex II [44]. Hence, these results suggested that the downregulation of both transcription and translation of mtDNA reduced adiposity and increased insulin sensitivity in mice.

Several studies have shown that mitochondria are key participants in innate immune pathways and important drivers of inflammation. Mild, transient perturbations to the mitochondrial ETC reduce inflammation in mice [45]. The nuclear transcription factor NFκB regulates immunity by controlling the expression of related inflammatory genes. It has been demonstrated that NFκB plays an essential role in inflammatory responses associated with insulin resistance in genetic mutant mice [4]. In this study, we found that *Mtfmt* deletion decreased the NFκB activity and inflammatory response induced by LPS in macrophages. This was consistent with the findings of animal experiments that Het mice could resist metabolic inflammation induced by HFD. Seo et al. showed that, under basal conditions, NFκB activity was decreased in *Mtfmt*-silenced Hela cells with defective mitochondria, which may explain their reduced ability to defend against intracellular infection in the early stages of infection [46]. However, the effect of *Mtfmt* deletion on NFκB activity has never been experimentally proved. The elevation of mROS contributes to increased secretion of systemic inflammatory factors via the activation of NFκB [18,47]. In this study, we found that *Mtfmt* knockdown in macrophages decreased mitochondrial activity and the production of mROS. Thus, we suggested that *Mtfmt* knockdown in macrophages reduced mROS production and led to reduced activation of NFκB. Hence, *Mtfmt* knockdown in macrophages reduced mROS production and led to blunted NFκB activation, which further led to downregulation of the levels of relevant inflammatory factors and consequently improved the metabolic impairment in mice.

In conclusion, *Mtfmt* deficiency alleviated HFD-induced metabolic disorders. The reduction in *Mtfmt* levels in macrophages reduced mitochondrial activity and mROS production, which decreased NFκB activation and, in turn, affected inflammation. These data demonstrated that targeting *Mtfmt*-mediated mitochondrial protein translation to regulate metaflammation might be an innovative therapeutic option for treating metabolic diseases.

## 4. Materials and Methods

### 4.1. Animals

According to the structure of the *Mtfmt* gene, exon 2-exon 4 of the *Mtfmt*-201 (ENSMUST00000074792.6) transcript is recommended as the knockout region. The region contains 436 bp coding sequences. Knocking out the region will result in the disruption of protein function. A brief summary of the procedure is as follows: sgRNA was generated in vitro. Fertilized C57BL/6 mouse eggs were microinjected with Cas9 and sgRNA. The F0 mice were obtained by transplanting fertilized eggs, which were confirmed by PCR and sequencing. By mating positive F0 generation mice with C57BL/6 wild-type (WT) mice, a stable F1 generation mouse model was obtained. Tails from the pups and embryo samples were obtained after mating with C57BL/6 WT mice, and genotyping was carried out on a 96-well thermal cycler (Thermal Cycler PTC0200, Bio-Rad, Hercules, CA, USA), utilizing two distinct amplification reactions for each mouse using two primer sets. In these two pairs of primers, FI and RI are located outside the knockout fragment, and F2 and R2 are located inside the knockout fragment, so the genotype of the mouse or embryo can be determined according to the size of the amplified PCR product fragment. The forward and reverse sequences were as follows: F1, 5′-AAAGTTCGTCCCTTCCTGGTG-3′ and R1, 5′-TTACTTCAGAGGTGGTTGGCAG-3′ (primer 1); F2, 5′-ATCGAACTCCTTGGCTTTCCTAC-3′ and R2, 5′-CATAATGGACTGGACATGGGAC-3′ (primer 2). PCR amplification was performed under the following conditions: 95 °C for 5 min, followed by 20 cycles of 98 °C for 30 s, 65 °C (decreased 0.5 °C each cycle) for 30 s, and 72 °C for 45 s, then followed by 20 cycles of 98 °C for 30 s, 55 °C for 30 s, and 72 °C for 45 s, then 1 cycle at 72 °C for 5 min. To characterize embryonic lethality, embryos were harvested at 13.5 dpc and small pieces were genotyped. As described above, the PCR conditions were the same. Later experiments were conducted with *Mtfmt* heterozygous knockout (Het KO) male mice, since homozygous knockout mice exhibited embryonic lethality. 

The WT and Het KO male mice were normally housed in standard cages with free access to food and water under a 12 h dark–light cycle for 28 weeks. The 4-week-old littermates of the WT and Het KO male mice were randomly assigned to feed on either the low-fat diet (LFD, 10% kcal fat; XTCON50J, Jiangsu Xietong Pharmaceutical Bio-engineering Co., Ltd., Nanjing, China) or the high-fat diet (HFD, 60% kcal fat; XTHF60, Jiangsu Xietong Pharmaceutical Bio-engineering Co., Ltd., Nanjing, China) for 17 weeks. The Nanjing Agricultural University Institutional Animal Care and Use Committee (IACUC) approved all experimental protocols, and all procedures followed the “Guidelines on Ethical Treatment of Experimental Animals.” (2006) No. 398 set by the Ministry of Science and Technology, China, and the “Regulation regarding the Management and Treatment of Experimental Animals” (2008) No. 45 set by the Jiangsu Provincial People’s Government.

### 4.2. Real-Time Polymerase Chain Reaction (RT-PCR)

TRIzol reagent (Invitrogen, Carlsbad, CA, USA) was used to isolate total RNA from Kupffer cells and tissues, which was then reverse-transcribed to cDNA using random hexamer primers (Promega, Madison, WI, USA). The real-time PCR was conducted with diluted cDNA (1:20, *v*/*v*) using the Mx3000P Real-time Polymerase Chain Reaction (PCR) System (Stratagene). The reference gene used was glyceraldehyde-3-phosphate dehydrogenase (GAPDH). Tsingke (Nanjing, China) synthesized all primers. The primer sequences for qPCR are listed in Supplementary Table S1.

### 4.3. Western Blot Analysis

The Western blot analysis was carried out as per standard protocols on 10% SDS/PAGE gels and then transferred to nitrocellulose membranes. The membranes were blocked in TBST with 0.1% Tween-20 and 5% non-fat dry milk for 2 h and then incubated with primary antibodies against Mtfmt (1:1000, cat. no.: PAH614Mu01, Cloud-Clone Corp, Houston, TX USA), p-IkBα (1:1000, cat. no.: 2859S, Cell Signaling, Danvers, MA, USA), IkBα (1:1000, cat. no.: 9242, Cell Signaling, Danvers, MA, USA), IL-1β (1:1000, cat. no.: ab254360, abcam, Cambridge, UK), SDHA (1:1000, cat. no.: 14865-1-AP, proteintech, Rosemont, IL, USA), VDAC (1:1000, cat. no.: 10866-1-AP, proteintech, Rosemont, IL, USA), ND6 (1:1000, cat. no.: BS1632, Bioworld, Nanjing, China), COX1 (1:1000, cat. no.: BS70809, Bioworld, Nanjing, China), β-actin (1:20,000, cat. no.: AC026, Abclonal, Wuhan, China), and GAPDH (1:1000, cat. no.: MB001H, Bioworld, Nanjing, China).

### 4.4. Transmission Electron Microscopy

Embryos (13.5-day-old) were isolated from pregnant females and fixed in 2.5% glutaraldehyde. Embryos were dehydrated and embedded in Araldite. Ultrathin sections were cut and stained with uranyl acetate and osmium tetroxide. Sections were examined in a Hitachi SU8010 electron microscope operated at 80 kV.

### 4.5. Glucose Tolerance Test (GTT)

Mice fasted for 12 h received an intraperitoneal glucose injection of 2 g/kg body weight during GTT. Before glucose injection (0 min) and 15, 30, 60, and 120 min afterward, blood samples were collected from the tail vein. A glucose meter (ACCU-CHEK Active Blood Glucose Meter, Roche) was used to measure blood glucose concentration immediately.

### 4.6. Insulin Tolerance Test (ITT)

Insulin (0.75 IU/kg, Aladdin, CAS 12584-58-6) was administered intraperitoneally for ITT. Glucose concentrations were measured before insulin injection (0 min) and 15 min, 30 min, 60 min, and 90 min after insulin injection. A glucose meter was used to immediately measure the blood glucose concentrations in the mouse tail veins after blood samples were collected.

### 4.7. Histological Analysis

For histomorphological evaluation, we fixed fresh livers with 4% paraformaldehyde, dehydrated them, embedded them in paraffin, and then stained them with hematoxylin and eosin. The cross sections were examined under a microscope (BX63F OLYMPUS Micro Image System, OLYMPUS, Tokyo, Japan).

### 4.8. Serum Biochemical Measurement

Analyses of serum alanine aminotransferase (ALT) activity (H001), aspartate aminotransferase (AST) activity (H002), and triglyceride (TG, H201) were performed using an automatic biochemical analyzer (Hitachi 7020, HITACHI, Tokyo, Japan) and respective commercial assay kits purchased from Ningbo Medical System Biotechnology Co., Ltd. (Ningbo, China).

### 4.9. Detection of MDA, SOD, and TGs in Mouse Livers

Liver triglycerides were assayed using a triglyceride assay kit (GPO-POD; Applygen Technologies Inc., Beijing, China). A Lipid peroxidation (MDA, malondialdehyde) Assay Kit and Superoxide dismutase (SOD) Activity Kit were purchased from Solarbio (Beijing, China). All detections were completed according to the manufacturer’s instructions.

### 4.10. Enzyme-Linked Immunosorbent Assay (ELISA)

ELISA kits were used to detect levels of interleukin 1β (IL-1β) and tumor necrosis factor α (TNFα) (Cusabio, cat. no.: CSB-E08054m, CSB-E04741m, Wuhan, China), following the manufacturer’s protocols. Briefly, standards or samples were added to micro-ELISA strip plate wells and combined with specific antibodies. Antibodies conjugated to horseradish peroxidase (HRP) were added to each well, and free components were then washed away. TMB substrate solution was added to each well. The optical density (OD) of each sample was measured spectrophotometrically at 450 nm, and the concentration was determined by comparing the OD of each sample with the standard curve.

### 4.11. Cell Culture and Cell Transfection 

Kupffer cells (BeNa Culture Collection, Kunshan, China, BNCC340733) were cultured at 37 °C in a 5% CO_2_ atmosphere in 1640 medium (Wisent, cat. no.: 350-000-CL, Nanjing, China) containing 10% (*v*/*v*) fetal bovine serum. During 70% confluent growth, Kupffer cells were treated for 6 or 12 h with 100 ng/mL LPS [48]. Using jetPRIME^®^ transfection reagent (Polyplus Transfection, Beijing, China), specific small interfering RNAs (*Mtfmt* siRNA1, *Mtfmt* siRNA2, and *Mtfmt* siRNA3; GenePharma, Shanghai, China) were transfected into Kupffer cells to knock down *Mtfmt*. The scramble siRNA served as a negative control (NC siRNA). The sequences are listed in Supplementary Table S2.

### 4.12. Flow Cytometry Analysis

To measure mitochondrial membrane potential, we incubated the cells with complete media containing 2.5 μM JC-1 dye (Thermo Fisher, cat. no.: T3168, Waltham, MA, USA), then harvested them with FACS buffer (2% FBS in phosphate-buffered saline) and analyzed them with flow cytometry (BD Biosciences, San Jose, CA, USA) after 30 min at 37 °C.

### 4.13. Immunofluorescence 

To fix Kupffer cells, 4% paraformaldehyde was used for 10 min. Each section was treated with Tris-buffered saline containing 0.3% Triton X-100 for 1 h, blocked with 5% Bovine serum albumin, and then exposed to the primary antibody for an overnight incubation at 4 °C before being exposed to the secondary antibody. The cell nuclei were marked with DAPI.

### 4.14. Mitochondrial ROS Determination

Kupffer cells were transfected with control or Mtfmt siRNA and treated with 1 μg/mL LPS [49] for 30 min, and then cell samples were stained with 5 μM MitoSOX red mitochondrial superoxide indicator (Thermo Fisher, cat. no.: M36008, Waltham, MA, USA) for 10 min at 37 °C in order to detect mitochondrial ROS. Three HBSS washes were performed on the labeled cells. Using a Zeiss Observer, which is an inverted microscope, fluorescence images were captured (Carl Zeiss, Thornwood, NY, USA).

### 4.15. Isolation of Mitochondria

Mitochondrial isolation from cultured Kupffer cells was performed using a commercial Mitochondria Isolation Kit (Solarbio, cat. no.: SM0020, Beijing, China), as per the manufacturer’s protocol. Briefly, a total of 1 mL of precooled lysis buffer was used to resuspend Kupffer cells collected by trypsinization. In an ice bath, the cell suspensions were ground 30 times in a small-volume glass homogenizer. After centrifugation at 1000× *g* at 4 °C for 5 min, performed twice, the supernatants were further centrifuged at 12,000× *g* at 4 °C for 10 min to obtain the crude mitochondrial precipitates. The mitochondrial precipitates were resuspended in 50 μL of wash buffer, then centrifuged at 4 °C for 5 min at 1000× *g*. The supernatants were centrifuged at 12,000× *g* for 10 min at 4 °C to obtain mitochondrial precipitates of high purity. These obtained mitochondrial precipitates were resuspended in store buffer or used immediately.

### 4.16. Statistical Analysis

GraphPad Prism 9 was used to analyze all data as means ± SEMs. To evaluate the normality of the distribution of values, Kolmogorov–Smirnov testing was employed for each variable. Two-way ANOVA with uncorrected Fisher’s LSD and unpaired Student’s *t*-tests were used. The differences were considered statistically significant when *p* < 0.05.

## Data Availability

All data supporting the findings of this study are available from the corresponding author on reasonable request.

## Supplementary Materials

The supporting information can be downloaded at: https://www.mdpi.com/article/10.3390/ijms24065999/s1.
