# Supplementary material for: Mitochondrial Methionyl-tRNA Formyltransferase Deficiency Alleviates Metaflammation by Modulating Mitochondrial Activity in Mice
Source: Int J Mol Sci. 2023 Mar 22;24(6):5999. doi: 10.3390/ijms24065999 (PMC10051599; doi:10.3390/ijms24065999)
Supplement: Supplementary file 1 [file ijms-24-05999-s001.zip › ijms-2218072-supplementary.pdf]

Mitochondrial Methionyl-tRNA Formyltransferase Deficiency Alleviates Metaflammation by Modulating Mitochondrial Activity in Mice

Xiaoxiao Sun [1], Suyuan Liu [1], Jiangxue Cai [1], Miaoxin Yang [1], Chenxuan Li [1], Meiling Tan [1] and Bin He [1,2,*]

[1] Key Laboratory of Animal Physiology & Biochemistry, Ministry of Agriculture and Rural Affairs, College of Veterinary Medicine, Nanjing Agricultural University, Nanjing 210095, China
[2] MOE Joint International Research Laboratory of Animal Health & Food Safety, Nanjing Agricultural University, Nanjing 210095, China
* Correspondence: heb@njau.edu.cn; Tel.: +86-25-84396413

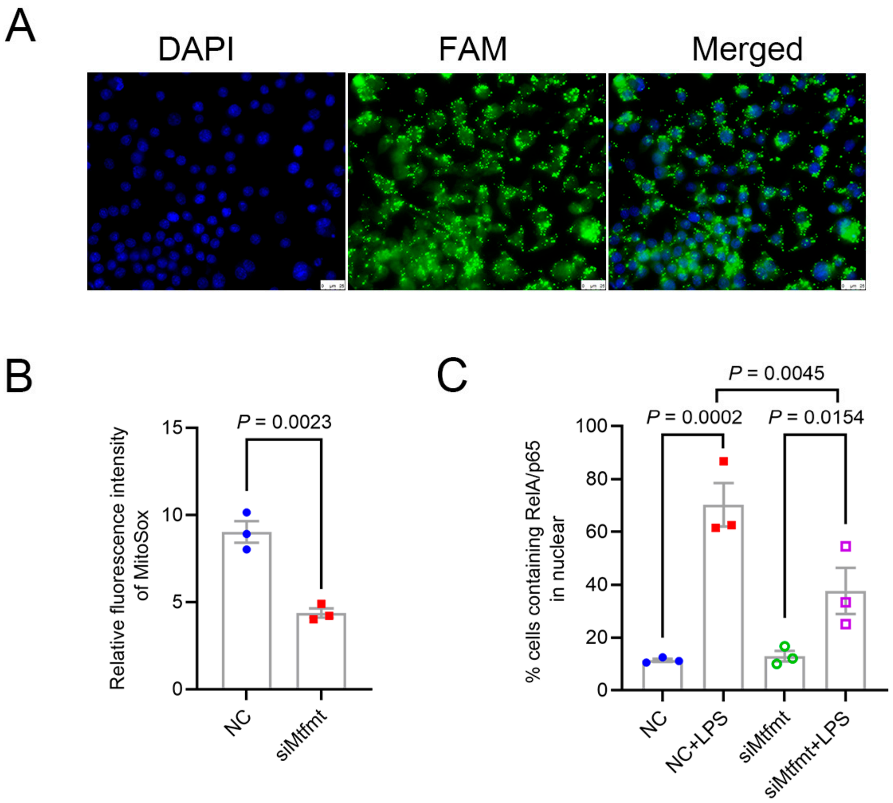

Figure S1. (A) Fluorescent images of Kupffer cells after 6 hours of transfection with siRNA conjugated FAM. (B) Quantification of fluorescence intensity of MitoSOX, n=3. (C) Quantification of P65 nuclear localization, n=3.

Table S1. The primer sequences of the target genes

Gene	Forward	Reverse
Mtfmt	TCGCTATCCCCCAAAGGACT	CACTCAAAAGCCGGCCAAAG
IL-1β	CTTCAGGCAGGCAGTATC	CAGCAGGTTATCATCATCATC
IL-6	GACAAAGCCAGAGTCCTTCAGA	TGTGACTCCAGCTTATCTCTTGG
TNFα	GACGTGGAACTGGCAGAAGA	ACTGATGAGAGGGAGGCCAT
ND6	TATTGCCGCTACCCCAATCC	GGGGGATGTTGGTTGTGTTTG
ND1	CGTCCCCATTCTAATCGCCA	ATGGCGTCTGCAAATGGTTG
COX1	TCGGAGCCCCAGATATAGCA	TTTCCGGCTAGAGGTGGGTA
GAPDH	ACCCTTAAGAGGGATGCTGC	CCCAATACGGCCAAATCCGT

Table S2. The sequences of *Mtfmt* siRNA

siRNA	Sense	Antisense
Mtfmt siRNA 1	GAGGCUCUUAUUCUUAAAUTT	AUUUAAGAAUAAGAGCCUCTT
Mtfmt siRNA 2	GCGUAACGAUUAUGCAAAUTT	AUUUGCAUAAUCGUUACGCTT
Mtfmt siRNA 3	GCCCGAAAGUCUGAACAAUTT	AUUGUUCAGACUUUCGGGCTT
